# Exogenous plasmid capture to characterize tetracycline-resistance plasmids in sprouts obtained from retail in Germany

**DOI:** 10.3389/fmicb.2025.1538973

**Published:** 2025-02-05

**Authors:** Maria Stein, Erik Brinks, Diana Habermann, Gyu-Sung Cho, Charles M. A. P. Franz

**Affiliations:** Department of Microbiology and Biotechnology, Max Rubner-Institut, Federal Research Institute of Nutrition and Food, Hermann-Weigmann, Kiel, Germany

**Keywords:** extrachromosomal DNA, plasmid, sprout, antibiotic resistance, conjugation, metagenomic

## Abstract

This study aimed to characterize antibiotic-resistance plasmids present in microorganisms from sprout samples using exogenous plasmid capture. Fresh mung bean sprouts were predominantly colonized by bacteria from the phyla *Proteobacteria* and *Bacteroidetes*. To capture plasmids, a plasmid-free *Escherichia* (*E.*) *coli* CV601 strain, containing a green fluorescent protein gene for selection, was used as the recipient strain in exogenous plasmid capture experiments. Transconjugants were selected on media containing cefotaxime or tetracycline antibiotics. While no cefotaxime-resistant transconjugants were obtained, 40 tetracycline-resistant isolates were obtained and sequenced by Illumina NextSeq short read and Nanopore MinION long read sequencing. Sequences were assembled using Unicycler hybrid assembly. Most of the captured long plasmids carried either the *tet*(A) or *tet*(D) resistance gene, belonged to the IncFI or IncFII replicon types, and were predicted as conjugative. While the smaller plasmids contained the *tet*(A) tetracycline resistance gene as well as additional quinolone (*qnr*S1), sulfonamide (*sul*1) and trimethoprim (*dfr*A1) resistance genes, the larger plasmids only contained the *tet*(D) resistance gene. An exception was the largest 192 kbp plasmid isolated, which contained the *tet*(D), as well as sulfonamide (*sul1*) and streptomycin (*aad*A1) resistance genes. The smaller plasmid was isolated from different sprout samples more often and showed a 100% identity in size (71,155 bp), while the 180 kbp plasmids showed some smaller or larger differences (in size between 157,683 to 192,360 bp). This suggested that the plasmids obtained from the similar sprout production batches could be clonally related. Nanopore MinION based 16S metagenomics showed the presence of *Enterobacter* (*En.*) *cloacae*, *En. ludwigii*, *En. kobei*, *Citrobacter* (*C.*) *werkmanii*, *C. freundii*, *Klebsiella* (*K.*) *oxytoca* and *K. pneumonia*, which have previously been isolated from fresh produce in Germany. These bacteria may harbor antibiotic resistance genes on plasmids that could potentially be transferred to similar genera. This study demonstrated that bacteria present in sprouts may act as the donors of antibiotic resistance plasmids which can transfer resistance to other bacteria on this product via conjugation.

## Introduction

Antibiotic resistance of pathogenic bacteria is a major threat to public health, for which a global strategy is necessary to control the problem (Antimicrobial Resistance, 2022). Currently, antibiotic-resistant bacteria such as methicillin-resistant *Staphylococcus* (*S.*) *aureus*, carbapenem-resistant *Acinetobacter* (*A.*) *baumannii* and *Klebsiella* (*K.*) *pneumoniae*, fluoroquinolone resistant *Escherichia* (*E.*) *coli* and third generation cephalosporin-resistant *E. coli* and *K. pneumonia* are particularly problematic (Antimicrobial Resistance, 2022). Antibiotic resistant bacteria are ubiquitous and the living areas of humans and animals are interwoven with the environment, so that there are no borders for the transfer of microorganisms and antibiotic resistance genes in nature ecosystems (Aminov, 2011). The ‘One Health’ approach thus aims to attain an encompassing optimal health for people, domestic animals, wildlife, plants, and our environment. It also seeks to prevent the occurrence and spread of antibiotic resistances, thereby preserving the continued effectiveness of existing antimicrobials (McEwen and Collignon, 2018; Collignon and McEwen, 2019).

The consumption of fruit and vegetables, including salads and sprouts, is important for a balanced and healthy diet, as these supply a combination of vitamins, antioxidants and minerals. Based the thermolabile characteristics of plant foods and their constituents, these are often consumed either only minimally processed or raw. This can be a problem if pathogenic bacteria occur in these products, as such bacteria would not be reduced by an appropriate inactivation processing step such as heating. Apart from meats and meat products, fresh produce and plant food products are also known to contain pathogenic bacteria (Lynch et al., 2009; Buchholz et al., 2011; Olaimat and Holley, 2012; Callejon et al., 2015; Herman et al., 2015; Fiedler et al., 2017; Canning et al., 2023). Such plant food products, i.e., fresh spinach, ready-to-eat salad, packaged leafy green salad, sprout and cucumbers, have previously led to outbreaks with *E. coli* O157:H7 or O104:H4, *Salmonella enterica* subsp. *enterica* Serovar Coeln or *Listeria* (*L.*) *monocytogenes* (Buchholz et al., 2011; Sharapov et al., 2016; Vestrheim et al., 2016; Self et al., 2019). In Germany, sprouts were the cause of a large Shiga toxin-producing *E. coli* O104:H4 outbreak in 2011, which resulted in the deaths of 54 individuals (Buchholz et al., 2011; Frank et al., 2011). Furthermore, the German Federal Institute for Risk Assessment subsequently advised vulnerable consumers against the consumption of raw sprouts and fresh-cut salads (Bundesinstitut für Risikobewertung (2020)).

Apart from obligate human pathogens, fresh produce is also frequently contaminated with opportunistic pathogens. These products often show high numbers of *Enterobacteriaceae*, with contamination levels found to vary for specific product groups. Particularly sprouts show high contamination levels (Abadias et al., 2008; Leff and Fierer, 2013; Fiedler et al., 2017). *Enterobacteriaceae* may be involved in spoilage of fresh produce and grow on nutrients released by the cutting process of the salads during preparation of fresh cut products (Ragaert et al., 2007; Chris Baylis et al., 2011). This can lead to rapid spoilage and is, therefore, one reason for the relatively short shelf life of these products. Fresh produce such as carrots, cucumber, tomatoes, herbs, salads, mixed salads and sprouts have been known for years to potentially harbor not only pathogens, but also antibiotic-resistant *Enterobacteriaceae* (Boehme et al., 2004; Schwaiger et al., 2011; Blau et al., 2018; Cho et al., 2021). Additionally, several studies have specifically investigated the occurrence of extended spectrum beta-lactamase (ESBL)- producing bacteria in fresh produce and found their prevalence to be generally low (Nuesch-Inderbinen et al., 2015; Huizinga et al., 2018; Kaesbohrer et al., 2019). Additionally, some studies have focused on tetracycline and cefotaxime resistant *Enterobacteriaceae* from fresh produce, revealing the presence of strains with multiple resistances to tetracycline and cefotaxime as well as other antibiotics which lead to an extended spectrum, beta-lactamase resistance phenotype (Blau et al., 2018; Cho et al., 2021; Kläui et al., 2024). The development of multidrug resistant bacteria is believed to be due to the use of antibiotics in animal husbandry and the transfer of resistant strains from animals to the plant products through the use of manure as fertilizer (Heuer et al., 2011; Lima et al., 2020). In particular, tetracyclines are the most commonly used antibiotics employed for therapy of food-producing animals. Due to incomplete absorption antibiotics are excreted with manure and can accumulate in agricultural soil and be transferred to produce (Heuer et al., 2011; Jechalke et al., 2014). Sprouts, which on the other hand are grown in hydroculture, are able to become contaminated with antibiotic-resistance bacteria through several routes, including contaminated seeds, irrigation water, or workers involved in sprout production (Boehme et al., 2004; Iseppi et al., 2018).

Bacterial plasmids can play a role in the dissemination of antibiotic resistance genes (von Wintersdorff et al., 2016; Jian et al., 2021). Therefore, investigations on antibiotic resistances in bacteria from fresh produce have so far also focused on plasmids, using PCR typing or short read sequencing to detect plasmids with various Inc. types such as IncFIA, IncFIB, IncFII, IncHI1, IncHI2, IncI1, IncI2, IncK, IncL/M, IncN, IncP, IncQ1, IncR, IncU, IncX, IncY and Col replicon types (Blau et al., 2018; Freitag et al., 2018; Huizinga et al., 2018; Iseppi et al., 2018; Cho et al., 2021). The assignment of resistance genes to such plasmids is often impossible, as the commonly used Illumina short read sequencing for whole genome sequencing cannot resolve the plasmid sequence in one continuous contig as a result of repetitive sequences. However, previous studies using conjugation experiments have shown that plasmids present in fresh produce can be transferred (Reuland et al., 2014; Blau et al., 2018; Freitag et al., 2018; Iseppi et al., 2018). Blau et al. (2018) demonstrated that exogenous plasmid capturing could be used to detect plasmids which contained tetracycline antibiotic resistance genes such as *tet*(A) and *tet*(Q) together with various Inc. types such as IncF, IncI and IncP in mixed salad, cilantro, and arugula. As plant produce is often consumed raw and can contain antibiotic resistance genes, such plasmids may thus act as vehicles to transfer antibiotic resistance genes to commensal bacteria in the gut.

This study aimed to capture predominant plasmids naturally present in the bacterial population of sprouts using the exogenous plasmid capturing technique (Blau et al., 2018) and to analyze these for the presence of antibiotic resistance genes. For this, the study aimed to completely sequence the captured plasmids by both long read and short read sequencing using MinION Nanopore and Illumina sequencing technologies, respectively, and to obtain the nucleotide sequence of the plasmids as a single contig. Furthermore, the study aimed to make sequence-based predictions as to the transferability of the plasmids present in sprouts.

## Materials and methods

### Bacterial strain and culture conditions

The *E. coli* strain CV601 (Heuer et al., 2012) was used as recipient strain in this study to capture exogenous plasmids according to Blau et al. (2020). Using this method, the donor strains remain unknown, as only the recipient strain colonies with the captured plasmids (transconjugants) are isolated after the exogenous capture experiments. *E. coli* CV601 was gratefully received from Prof. K. Smalla of the Julius Kühn Institute in Braunschweig, Germany, and was previously genetically modified to contain the green fluorescent protein (*gfp*) of the jellyfish *Aequorea victoria* to allow selection of the transconjugants after plasmid conjugation experiments using a UV light source (Smalla et al., 2000; Heuer et al., 2002). The strain is resistant to 50 μg/mL of either kanamycin or rifampicin but susceptible to tetracycline. *E. coli* CV601 was routinely grown in Luria Bertani (LB) broth (Lennox) (Carl Roth, Karlsruhe, Germany) containing 50 μg/mL of each kanamycin (Applichem PanReac, Darmstadt, Germany) and rifampicin (Applichem PanReac).

### Exogenous plasmid capture and transconjugant selection

Exogenous plasmid capture was performed on sprouts with the naturally present bacteria serving as plasmid donors. The recipient bacteria were prepared by inoculating 1% of an overnight culture of *E. coli* CV601 into 20 mL of LB broth in an Erlenmeyer flask and incubating at 37°C with shaking at 130 rpm. After 7 h incubation, a further 20 mL of LB broth containing 50 μg/mL of each kanamycin and rifampicin was inoculated with 1% inoculum from fresh culture without antibiotics and then incubated overnight at 37°C with shaking at 130 rpm. Cells were harvested by centrifugation (5.000 x*g*, 5 min), and the pellet was washed twice with 10 mM Tris and then resuspended in 20 mL of quarter-strength Ringer’s solution (Merck, Darmstadt, Germany).

Exogenous plasmid experiments were done on four separate sampling occasions (SP1, SP2, SP3 and SP4; SP = sprout product) within the time frame of ca. 3 weeks, using a sprout product from the same producer. Products were purchased from a local supermarket. Two samples of sprout were taken from the same package on each sampling occasion. Thus, four sprout products, each obtained at a different date from retail, were sampled each in technical duplicates (eight samples in total). For the exogenous plasmid transfer, 10 g of a retail sprout mix (alfalfa and red radish sprouts) were weighed into a 50 mL screw cap Falcon tube and 1 mL of the washed overnight culture of *E. coli* CV601 was added to the sprout mix. The tubes were vortexed for 1 min to distribute the bacteria onto the sprouts and were then incubated at 37°C for 22 h. The screw caps were only loosely tightened to allow air transfer and enable aerobic growth of the recipient *E. coli* CV601. After the conjugation, 10 mL of a 0.85% NaCl solution was added to the tube and bacterial cells were suspended by vortexing. The liquid was withdrawn as much as possible from the tube and then centrifuged at 5.000 x*g* for 10 min and the pellet was next resuspended in 1 mL of 0.85% NaCl. Cells were then diluted in a tenfold dilution series using quarter-strength Ringer’s solution and aliquots of 100 μL of relevant dilutions were plated onto selective agar plates in duplicate. In addition, either 100 μL or 300 μL of the undiluted bacteria were also plated out in duplicate on 90 mm or 145 mm agar plates, respectively. The selective agar plates consisted of LB agar containing 50 μg/mL of each kanamycin, rifampicin and either 50 μg/mL tetracycline (Sigma Chemie, Steinheim, Germany) or 10 μg/mL cefotaxime (Sigma Aldrich, Taufkirchen, Germany). While rifampicin and kanamycin were selective for the resistant recipient strain, tetracycline and cefotaxime were used to select recipients which captured plasmids harboring tetracycline or cefotaxime (possibly extended spectrum beta lactamase) resistance determinants. The selective agar plates were incubated at 37°C for up to 3 days.

To select transconjugants, the selective agar plates were examined for fluorescent colonies under UV light at 365 nm. Up to 20 colonies from each of the eight sprout samples were randomly picked from plates of the highest dilutions which showed well-separated colonies. This way we aimed to randomly select for the plasmids of the predominant bacteria present in the sprout samples. These were then streaked out again onto LB agar medium containing 50 μg/mL of each tetracycline, rifampicin and kanamycin. Plates were incubated at 37°C overnight. In addition, for a quick screening to determine whether the strains were indeed the recipient strain, a PCR determination was performed targeting the green fluorescence gene. For this, a colony from the plate was picked, suspended in 50 μL of distilled water, and then lysed by heating at 98°C for 10 min. Two microliters of the lysate were used as DNA template for the PCR using the primers CV601F (5′- AGT GCC ATG CCC GAA GGT TA-3′) and CV601R (5′- AGC CAT CAT GCC GTT CAA AGT-3′), which amplifies a 799-bp fragment of the *gfp* gene in this strain. The PCR reaction contained 2.5 μL of each of the primers (10 pM/μl), 2 μL DNA lysate, 5 μL iTaq™ Universal SYBR® Supermix, and 2.5 μL nuclease free water. DNA was amplified by initial heating at 95°C for 10 min, followed by 34 cycles of denaturation at 95°C for 30s, primer annealing at 60°C for 30s and extension at 72°C for 30s, followed by a final extension of 5 min at 72°C. PCR products were confirmed by electrophoresis of a 1.5% agarose gel containing 1 μL gel red at 100 V for 75 min. Of the 20 initially selected colonies per 4 duplicate sprout samples, five colonies were finally selected, so that a total of 40 different transconjugants were obtained for whole genome sequencing. All transconjugants were randomly selected regarding the plasmid they contained, but using UV light and PCR, it was confirmed that they represented the recipient strain used in the study.

### Phenotypic antibiotic resistance testing of transconjugants

The phenotypic antibiotic resistance of all selected transconjugants was tested using the disk diffusion method according to the Clinical and Laboratory Standards Institute (CLSI) standard (Clinical and Laboratory Standards Institute, 2022). Antibiotics used included meropenem (MEM), tetracycline (TE), chloramphenicol (CHL), streptomycin (S), trimethoprim/sulfamethoxazol (SXT) and ciprofloxacin (CIP) (Table 1).

**Table 1:** Genomic characterization of extrachromosomal DNA of 40 transconjugants isolated in this study.

**Isolate** ^**1**^	Plasmid (Accession no.)	**Sequence length (bp)**	**mol% GC content**	**Complete circular**	**Inc/replicon type** ^**2**^	**Resistance gene(s)** ^**2**^	**Predicted mobility type (MPF type)** ^**2**^
X/SP1-CαI/2	pTEC11_3 (PQ667064)	71,155	53.69	yes	IncFII(pCRY)	*qnr*S1, *sul*1, *dfr*A1, *tet*(A)	Conjugative (MPF_I_)
X/SP1-CαI/7		71,155	53.69	yes	IncFII(pCRY)	*qnr*S1, *sul*1, *dfr*A1, *tet*(A)	Conjugative (MPF_I_)
X/SP1-CαI/11		71,155	53.69	yes	IncFII(pCRY)	*qnr*S1, *sul*1, *dfr*A1, *tet*(A)	Conjugative (MPF_I_)
X/SP1-CαI/13	pTCE14_2 (PQ667065)	149,450*	50.96	yes			
		71,155	53.69	yes	IncFII(pCRY)	*qnr*S1, *sul*1, *dfr*A1, *tet*(A)	Conjugative (MPF_I_)
X/SP1-CαI/19		71,155	53.69	yes	IncFII(pCRY)	*qnr*S1, *sul*1, *dfr*A1, *tet*(A)	Conjugative (MPF_I_)
X/SP1-CαII/2		71,155	53.69	yes	IncFII(pCRY)	*qnr*S1, *sul*1, *dfr*A1, *tet*(A)	Conjugative (MPF_I_)
X/SP1-CαII/3		71,155	53.69	yes	IncFII(pCRY)	*qnr*S1, *sul*1, *dfr*A1, *tet*(A)	Conjugative (MPF_I_)
X/SP1-CαII/5		71,155	53.69	yes	IncFII(pCRY)	*qnr*S1, *sul*1, *dfr*A1, *tet*(A)	Conjugative (MPF_I_)
X/SP1-CαII/6		71,155	53.69	yes	IncFII(pCRY)	*qnr*S1, *sul*1, *dfr*A1, *tet*(A)	Conjugative (MPF_I_)
X/SP1-CαII/7		71,155	53.69	yes	IncFII(pCRY)	*qnr*S1, *sul*1, *dfr*A1, *tet*(A)	Conjugative (MPF_I_)
X/SP2-CαI/1		71,155	53.69	yes	IncFII(pCRY)	*qnr*S1, *sul*1, *dfr*A1, *tet*(A)	Conjugative (MPF_I_)
X/SP2-CαI/5	pTCE52_3(PQ667066)	180,007	52.04	yes	IncFIB(K)	*tet*(D)	Conjugative (MPF_I_)
	4,624	43.73	yes	Col440I	Non-mobilizable	
	3,574	43.84	yes	Col(pHAD28)	Non-mobilizable	
X/SP2-CαI/8	pTCE53_2(PQ667067)	179,751	52.02	yes	IncFIB(K)	*tet*(D)	Conjugative (MPF_F_)
	3,574	43.84	yes	Col(pHAD28)	Non-mobilizable	
X/SP2-CαI/13	pTCE54_2(PQ667068)pTCE54_5(PQ667069)	192,360	52.78	yes	IncFIB(K), IncFII(K)	*tet*(D)*, sul*1, *aad*A1	Conjugative (MPF_F_)
	4,052	53.65	yes	Col440II	mobilizable	
X/SP2-CαI/19		71,155	53.69	yes	IncFII(pCRY)	*qnr*S1, *sul*1, *dfr*A1, *tet*(A)	Conjugative (MPF_I_)
X/SP2-CαII/1	pTCE56_19(PQ667070)	71,035	53.72	yes	IncFII(pCRY)	*qnr*S1, *sul*1, *dfr*A1, *tet*(A)	Conjugative (MPF_I_)
X/SP2-CαII/7		71,155	53.69	yes	IncFII(pCRY)	*qnr*S1, *sul*1, *dfr*A1, *tet*(A)	Conjugative (MPF_I_)
X/SP2-CαII/13		71,155	53.69	yes	IncFII(pCRY)	*qnr*S1, *sul*1, *dfr*A1, *tet*(A)	Conjugative (MPF_I_)
X/SP2-CαII/16		71,155	53.69	yes	IncFII(pCRY)	*qnr*S1, *sul*1, *dfr*A1, *tet*(A)	Conjugative (MPF_I_)
X/SP2-CαII/19		71,155	53.69	yes	IncFII(pCRY)	*qnr*S1, *sul*1, *dfr*A1, *tet*(A)	Conjugative (MPF_I_)
X/SP3-CαI/3		71,155	53.69	yes	IncFII(pCRY)	*qnr*S1, *sul*1, *dfr*A1, *tet*(A)	Conjugative (MPF_I_)
X/SP3-CαI/7		71,155	53.69	yes	IncFII(pCRY)	*qnr*S1, *sul*1, *dfr*A1, *tet*(A)	Conjugative (MPF_I_)
X/SP3-CαI/11	pTCE63_4(PQ667071)pTCE63_7(PQ667072)pTCE63_9(PQ667073)	157,683	52.21	no		*tet*(D)	Conjugative (MPF_F_)
	10,102	46.27	no	IncFIB(K)	Non-mobilizable	
	4,624	43.73	yes	Col440I	Non-mobilizable	
	3,574	43.84	yes	Col(pHAD28)	Non-mobilizable	
X/SP3-CαI/14	pTCE64_3(PQ667074)pTCE64_5(PQ667075)XXXXXX	180,007	52.04	yes	IncFIB(K)	*tet*(D)	Conjugative (MPF_F_)
	113,260	50.54	yes		Non-mobilizable	
	4,624	43.73	yes	Col440I	Non-mobilizable	
	3,574	43.84	yes	Col(pHAD28)	Non-mobilizable	
X/SP3-CαI/18		71,155	53.69	yes	IncFII(pCRY)	*qnr*S1, *sul*1, *dfr*A1, *tet*(A)	Conjugative (MPF_I_)
X/SP3-CαII/1		71,155	53.69	yes	IncFII(pCRY)	*qnr*S1, *sul*1, *dfr*A1, *tet*(A)	Conjugative (MPF_I_)
X/SP3-CαII/6		71,155	53.69	yes	IncFII(pCRY)	*qnr*S1, *sul*1, *dfr*A1, *tet*(A)	Conjugative (MPF_I_)
X/SP3-CαII/13	pTCE68_10(PQ667076)	71,047	53.67	yes	IncFII(pCRY)	*qnr*S1, *sul*1, *dfr*A1, *tet*(A)	Conjugative (MPF_I_)
X/SP3-CαII/16		71,155	53.69	yes	IncFII(pCRY)	*qnr*S1, *sul*1, *dfr*A1, *tet*(A)	Conjugative (MPF_I_)
X/SP3-CαII/20		71,155	53.69	yes	IncFII(pCRY)	*qnr*S1, *sul*1, *dfr*A1, *tet*(A)	Conjugative (MPF_I_)
X/SP4-CαI/3		180,007	52.04	yes	IncFIB(K)	*tet*(D)	Conjugative (MPF_F_)	
	4,624	43.73	yes	Col440I	Non-mobilizable		
	3,574	43,84	yes	Col(pHAD28)	Non-mobilizable	
X/SP4-CαI/7	pTCE72_2(PQ667077)	179,659	52.04	yes	IncFIB(K)	*tet*(D)	Conjugative (MPF_F_)
	4,624	43.73	yes	Col440I	Non-mobilizable	
	3,574	43.84	yes	Col(pHAD28)	Non-mobilizable	
X/SP4-CαI/9	pTCE73_2(PQ667078)pTCE73_4(PQ667079)	168,248	51.89	no	IncFIB(K)	*tet*(D)	Conjugative (MPF_F_)
	9,731	54.62	yes		Non-mobilizable	
	4,624	43.73	yes	Col440I	Non-mobilizable	
X/SP4-CαI/14		179,751	52.04	yes	IncFIB(K)	*tet*(D)	Conjugative (MPF_F_)	
	4,624	43.73	yes	Col440I	Non-mobilizable		
	3,574	43.84	yes	Col(pHAD28)	Non-mobilizable	
X/SP4-CαI/19		180,007	52.04	yes	IncFIB(K)	*tet*(D)	Conjugative (MPF_F_)
X/SP4-CαII/2		180,007	52.04	yes	IncFIB(K)	*tet*(D)	Conjugative (MPF_F_)	
	4,624	43.73	yes	Col440I	Non-mobilizable		
	3,574	43.84	yes	Col(pHAD28)	Non-mobilizable	
X/SP4-CαII/5		180,007	52.04	yes	IncFIB(K)	*tet*(D)	Conjugative (MPF_F_)	
	4,624	43.73	yes	Col440I	Non-mobilizable		
	3,574	43.84	yes	Col(pHAD28)	Non-mobilizable	
X/SP4-CαII/9		180,007	52.04	yes	IncFIB(K)	*tet*(D)	Conjugative (MPF_F_)	
	4,624	43.73	yes	Col440I	Non-mobilizable		
	3,574	43.84	yes	Col(pHAD28)	Non-mobilizable	
X/SP4-CαII/13		180,007	52.04	yes	IncFIB(K)	*tet*(D)	Conjugative (MPF_F_)	
	4,624	43.73	yes	Col440I	Non-mobilizable		
	3,574	43.84	yes	Col(pHAD28)	Non-mobilizable	
X/SP4-CαII/17	pTCE80_2(PQ667080)	181,207	52.01	yes	IncFIB(K)	*tet*(D)	Conjugative (MPF_F_)

* Indicates bacteriophage sequence; ^1^indicates the sample occasions 1–4 (SP1-SP4); ^2^was determined by bioinformatic analyses using ResFinder, PlasmidFinder, and Mob-Suit pipelines.

For the disk diffusion test, the strains were first streaked out onto Müller-Hinton agar plates without antibiotics and incubated at 37°C overnight. A single colony was resuspended into 9 mL NaCl-peptone [8.5 g/L NaCl (Merck), 1 g/L peptone (Merck)] solution to reach a density corresponding to 0.5 on the McFarland scale. The bacterial suspension was applied to the surface of a Müller-Hinton agar plate using a sterile swab, after which the antibiotic disks were applied to the surface with a dispenser and plates were incubated at 35°C +/−2°C for 18 h. The zones of inhibition around the antibiotic disk were measured with a caliper and the susceptibility toward the antibiotics were determined according to the breakpoint values stipulated by CLSI (Clinical and Laboratory Standards Institute, 2022). *E. coli* ATCC 25922 was used as a control strain as suggested by CLSI (Clinical and Laboratory Standards Institute, 2022). All phenotypic antibiotic resistance tests were performed in duplicate and the mean diameters of inhibition zones are reported.

### Whole genome sequencing

Genomic DNA for short-read sequencing using the Illumina NextSeq sequencing platforms was extracted from 1.5 mL overnight culture in LB-broth using the PeqGold Bacterial DNA Kit (VWR). The concentration and quality of the extracted DNA was measured using a NanoDrop 2000c (Thermo Fisher Scientific, Bremen, Germany) and a Qubit 3 spectrophotometer (Invitrogen, Darmstadt, Germany). The Illumina DNA-Prep Kit was used for library preparation according to the manufacturer’s instructions. Fragment sizes of the libraries were checked using the D1000 Kit on the TapeStation 4,150 (Agilent, Waldbronn, Germany). The final library was sequenced using a NextSeq 500 using NextSeq 500/550 Mid Output Kit with 2× 149 cycles paired end sequencing. The NextSeq sequence data were obtained as FASTQ-files and a quality control and sorting into paired and unpaired reads was done using Trimmomatic pipeline (v. 0.39). Only the paired sequences were used for hybrid assembly in this study.

For long-read sequencing using the MinION sequencing platform, bacteria were first grown in 100 mL LB broth at 37°C for overnight with shaking at 130 rpm. Genomic DNA was isolated from 500 μL using the Genomic Micro AX Bacteria Gravity Kit (A&A Biotechnology, Gdynia, Poland) according to the manufacturer’s instructions. The DNA quality was assessed with the Nanodrop 2000c and if not sufficient, purity was increased using AMPure XP or Mag-Bind TotalPure NGS beads. The DNA concentration was assessed using the Qubit spectrophotometer. For sequencing, up to 500 ng of DNA was used with the ligation and barcoding kit SQK-NBD112.96 following the protocol NBA_9137_v112_revK_ 01Dec2021-minion. The DNA was sequenced using a R10.4 flow cell in the MinION MK1B sequencing platform (Oxford Nanopore Technologies). The electronic raw signals (FAST5-format) were translated during base calling using the Guppy-Software (v. 6.1.2) and were saved as FASTQ-Format. Data were sorted using the Guppy-software (v. 6.1.2) according to their adapters (demultiplexed) and the adapter was removed.

Both long-read and short-read data were then hybrid assembled together using Unicycler (v. 0.4.9b) with conservative mode and default parameters. After assembly, the data were saved as FASTA files and analyzed bioinformatically using dDDH (Meier-Kolthoff et al., 2014),^1^ PlasmidFinder,^2^ ResFinder,^3^ Mob-suite (Robertson and Nash, 2018), PHASTER^4^ (Arndt et al., 2016), annotated with Prokaryotic Genome Annotation Pipeline (PGAP; Tatusova et al., 2016), and illustrated using CGview (Stothard and Wishart, 2005) with pgap data. For plasmid comparison using pairwise alignment BLASTN was used (Altschul et al., 1990).

### Determination of sprout microbiota by 16S rRNA gene targeted metagenomics

To determine the biodiversity of bacteria, present on the sprout samples and to identify potential plasmid donors, 16S rRNA gene-based metagenomics was performed on each of the four sprout samples obtained from the retail market on different occasions. 16S rRNA-gene metagenomics was performed with MinION sequencing. The PCR primers were chosen so that the almost complete 16S rRNA gene (ca. 1.500 bp) (Frank et al., 2008), inclusive of all variable regions (V1 to V9) could be amplified.

In order to extract total DNA of microorganisms of the sprout samples, 25 g of the sprout mix were each placed aseptically into a stomacher bag together with 225 mL buffered peptone water and were homogenized for 2 min on intensity setting 3 in a stomacher (Interscience, France). A 10 mL sample was collected and centrifuged at 5000 x*g* for 10 min and the pellet was frozen at −20°C (non-enriched sprouts). The rest of the sample was incubated in the stomacher bag for 20–24 h at 37°C for enrichment, after which again it was homogenized in the stomacher as described above. One ml of the sample (enriched sprouts) was collected and centrifuged at 5000 x*g* for 10 min and the pellet was also frozen at −20°C. Microbial DNA of both non-enriched sprouts and enriched sprouts were isolated using the NucleoSpin Microbial DNA Mini kit (Machery-Nagel) according to the manufacturer’s instructions. The 16S rRNA gene PCR was performed as described by Borges et al. (2023) using the 27F.1 (5′-AGR GTT TGA TCM TGG CTC AG-3′) forward primer and the 1492r (5’-TAC CTT GTT ACG ACT T-3′) reverse primer to which specific barcodes and spacer sequences were attached for every sample (Kuske et al., 2006; Frank et al., 2008; Borges et al., 2023). The barcodes used were compatible with Oxford Nanopore sequencing technology (Krych et al., 2019) and thus the PCR products could be directly used for sequencing with the MinION technology. The PCR reaction for the amplification of the 16S rRNA genes from all eight samples contained 2.5 μL each of the respective primers at a concentration of 10 pMol/μl, 6 μL dNTP mix, 5 μL DNA template, 10 μL 5x *Taq* polymerase buffer, 0.5 μL (1 unit) Q5® High-Fidelity DNA polymerase and 23.5 μL nuclease-free water. Five μl nuclease-free water was used instead of DNA in a negative control. The PCR conditions included an initial denaturation step at 94°C for 3 min, followed by 32 cycles of denaturation at 94°C for 30 s, primer annealing at 56°C for 30 s and extension at 72°C for 1 min 10 s. This was followed by a final extension step at 72°C for 5 min.

PCR products were checked for obtaining products with ca. 1,500 bp size by electrophoresis at 100 V for ca. 60 min using 1.5% agarose gels and 1 x TAE buffer with 1 μL gel red nucleic acid stain. The quality of the PCR products was also checked using the NanoDrop 2000c. The fragment sizes of PCR products were also measured using the D5000 Kit on the TapeStation 4,150 and PCR products were then purified to remove DNA of unwanted sizes using Mag-Bind® TotalPure NGS magnetic beads in a two-step purification approach, using 0.45-fold and 0.35-fold volumes of beads relative to library volume to remove small and large DNA fragments, respectively. In the second purification step, a small number of beads predominantly bound to large DNA molecules, and the supernatant was then used as precleaned PCR products.

The DNA concentration of the cleaned PCR products was measured using the Qubit dsDNA BR Assay Kit on a multimode microplate reader (Biotek Synergy LX, Agilent, Waldbronn, Germany). Samples were equimolarly pooled prior to the ligation step using 100 ng of each sample. A total of 200 ng DNA of the pooled sample was used for library preparation with the ligation sequencing Kit SQK-LSK114 according to the manufacturer’s instructions (‘genomic-dna-by-ligation-sqk-lsk114-GDE_9161_v114_revL_29Jun2022-minion’). The sequencing itself was done using a MinION flow cell R10.4.1 for 16.5 h. Base calling of raw data was done using the GPU Guppy Base calling software (v.6.1.2) and the FASTQ-files were demultiplexed with regard to their barcodes with Porechop (v. 0.2.4). The Q-score was analyzed with Geneious Prime® 2023.0.4 and 81.8 to 83.2% of reads reached a Q-score of at least Q20, while 58.6 to 62.2% of generated reads reached a Q-score of at least Q30. Subsequently, the sequencing data were grouped into clusters using NanoCLUST [Nextflow (21.10.6); NCBI-Database 16S ribosomal RNA 2023-04-04; NCBI-Datenbase taxdb 2023-04-03] using standard parameters (Rodriguez-Perez et al., 2021). The Nanoclust results (nanoclust_out.txt) were imported into Excel and the relative abundance of genera were calculated based on the total clustered reads. The relative abundances of genera were then graphically depicted using RStudio with ggplot2-package.

## Results

### Plasmid capture and phenotypic resistance determination

In this study, no transconjugants were obtained on cefotaxime-containing agar medium when the capture experiment was conducted at 37°C for 22 h. However, at the same conditions, several transconjugants could be recovered from tetracycline-containing medium. From duplicate samples of the four sprout mixes used in the experiment (SP1, SP2, SP3 and SP4), a total of 40 tetracycline-resistant transconjugants (5 from each of the technical duplicates) were selected.

The phenotypic antibiotic resistances determined for the transconjugants by disk diffusion test showed that none were resistant toward mereponem, ciprofloxacin, chloramphenicol, cefpodoxime and streptomycin at the concentrations used. As could be expected based on the fact that the transconjugants were isolated from tetracycline-containing agar medium, most of the transconjugants showed resistance or at least an intermediate resistance toward tetracycline in the disk diffusion test, according to the resistance/intermediate resistance classification stipulated by CLSI guidelines (Results not shown). Of the 40 transconjugants, 15 (37.5%) transconjugants were sensitive toward the trimethoprim/sulfamethoxazole antibiotic combination, with the remainder being resistant to this antibiotic combination according to the CLSI guideline.

### Genotypic characterization

The genomes of the 40 randomly selected transconjugants containing captured plasmids were sequenced by both Illumina short-read sequencing and Nanopore long-read sequencing. The genome sequences of transconjugants showed 99.9 to 100% similarity to the reference genome of *E. coli* strain CV601 (GenBank acc. no. CP043213.1) in an *in-silico* DNA–DNA (dDDH) analysis (results not shown), indicating that the recipient strain was isolated as transconjugant as intended. Additionally, the presence of the *aph*(3′)-III gene conferring resistance to kanamycin in this strain was confirmed using ResFinder.

**Figure 1:**
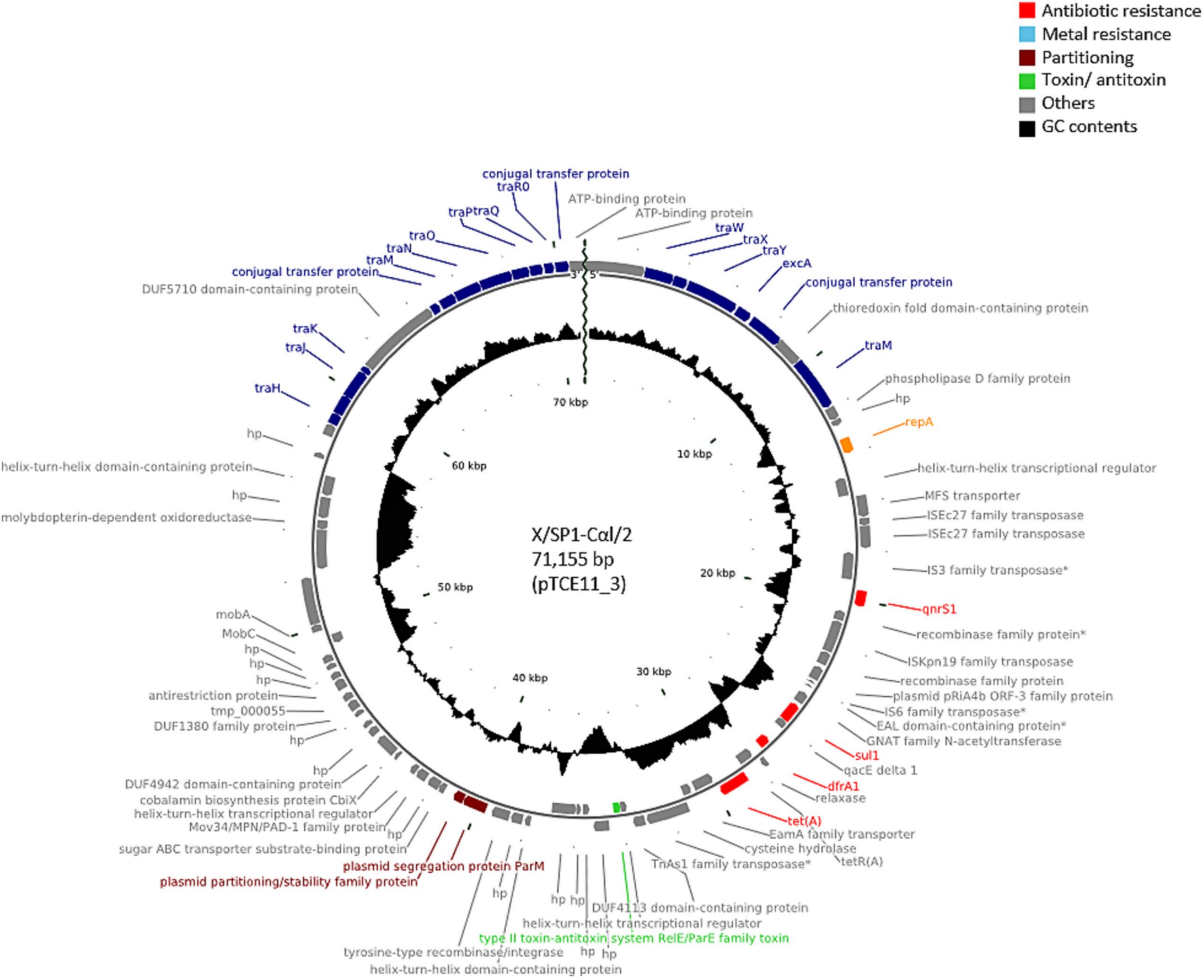
Plasmid map of the antibiotic-resistance plasmid pTCE11_3 from the recipient *E. coli* strain CV601. The plasmid map was created using CGview based on PGAP annotation data. hp., hypothetical protein. Antibiotic-resistance genes are indicated in red, and the conjugal proteins in blue.

PlasmidFinder was able to identify known replicon types in all of the transconjugants analyzed. In 25 of the transconjugants, plasmids containing the IncFII(pCRY) replicon type were present (Table 1), and these plasmids showed similar sizes of 71,035 bp (pTCE56_19), 71,047 bp (pTCE68_10), and 71,155 bp (*n* = 23). These plasmid sequences were all predicted to be circular and to contain the resistance genes to quinolones (*qnr*S1), sulfonamide (*sul*1), trimethoprim (*dfr*A1) and tetracycline [*tet*(A)]. When comparing the sequence similarity using BLASTN, the 23 plasmids of 71,155 bp were found to be identical. One of these plasmids presents in transconjugant X/SP1-CαI/2 (pTCE11_3) is shown in Figure 1. In addition to the antibiotic resistance genes, this plasmid was also found to contain a multiple facilitator superfamily (MFS) gene which encodes an efflux pump. Such MFS transporters are known to be associated with multidrug transport in enterobacteria (Kumar et al., 2013; Pasqua et al., 2021). It should be noted that one of these 25 transconjugants, i.e., X/SP1-CαI/13, apart from the 71,155 bp plasmid also possessed an additional extrachromosomal DNA fragment (Table 1; pTCE14_2), which PHASTER identified as the almost complete sequence (147,585 bp of 149,450 bp) of an intact (score = 141) prophage region possessing 281 CDS. This prophage sequence showed the highest similarity to the *Cronobacter* phage CR8 (NCBI acc. no. NC_024354.1; Table 1).

**Figure 2:**
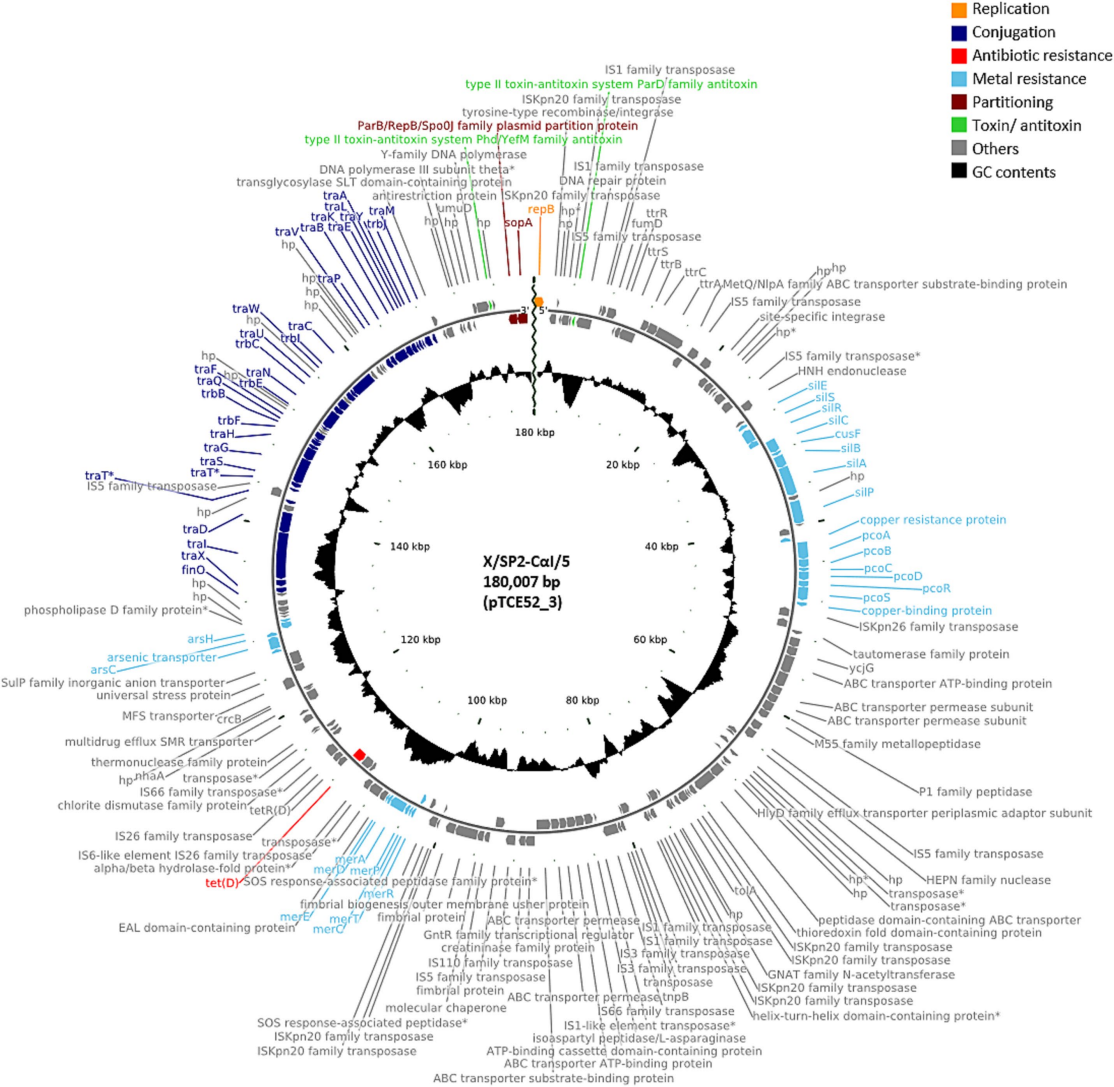
Plasmid map of the antibiotic-resistance plasmid pTCE52_3 from the recipient *E. coli* strain CV601. hp., hypothetical protein. Antibiotic-resistance gene is indicated in red, and the metal resistance genes and the conjugal proteins in light blue and dark blue, respectively.

A further 12 of the 40 randomly isolated transconjugants showed the presence of plasmids with a similar size of ca. 180,000 bp, i.e., 179,751 bp (n = 2; pTCE53_2), 179,659 bp (n = 1; pTCE72_2), 180,007 bp (n = 8; pTCE52_3), and 181,207 bp (n = 1; pTCE80_2). The structure of the most common plasmid of 180,007 bp (pTCE52_3) is shown in Figure 2. These had the same IncFIB(K) replicon type and harbored only the tetracycline resistance gene *tet*(D). Two transconjugants (X/SP4-CαI/19 and X/SP4-CαII/17) contained only the one large plasmid of ca. 180 kbp (pTCE75_2 and pTCE80_2, respectively), while the ten others additionally contained either a 3,574 bp plasmid (pTCE64_5) with a Col(pHAD28) replicon (only transconjugant X/SP2-CαI/8), or both the 3,574 bp Col(pHAD28) plasmid and the 4,624 bp Col440I plasmid (pTCE63_9) (Table 1). Another transconjugant (X/SP3-CαI/14) possessed 4 plasmids sequences, including a unique plasmid of 113,260 bp (pTCE64_3) that lacked both antibiotic resistance genes and an identifiable incompatibility type. Thus, it was classified as non-mobilizable (Table 1).

Among the three remaining of the 40 transconjugants, the transconjugants X/SP3-CαI/11 and X/SP4-CαI/9 each contained also a large contig of ca. 160 kbp (157,683 bp; pTCE63_4 and 168,248 bp; pTCE73_2, respectively), which could not be circularized. Transconjugant X/SP3-CαI/11 also contained a smaller contig of 10,102 bp (pTCE63_7) that contained the IncFIB(K) replicon that could also not be circularized (Table 1), while it also contained two contigs of 4,624 and 3,574 bp that were circularized and contained the Col440I and Col(pHAD28) replicons, respectively. Transconjugant X/SP4-CαI/9 on the other hand contained a smaller 9,731 bp (pTCE73_4) sequence that was circularized but possessed no known replicon type, as well as a 4,624 bp circularized sequence with the Col440I replicon type. The last transconjugant X/SP2-CαI/13 possessed the largest plasmid (192,360 bp; pTCE54_2) which harbored *tet*(D), *sul*1, and *aad*A1 resistance genes, as well as a 4,052 bp (pTCE54_5) plasmid with Col440II-replicon type. Using pairwise alignment, it could be shown that all 3,574 bp and all 4,624 bp plasmids showed 100% identity at the nucleotide level and that the 180,007 bp plasmids showed 99.98 to 100% identity.

Relating the plasmids to the relevant sprout samples in this study, the first investigated sprout sample (SP1) only allowed isolation of the 71,155 bp plasmid, while the last investigated sprout sample (SP4) allowed isolation of the large 180 kbp plasmids with the *tet*(D) gene in combination with the smaller Col plasmids. The sprout mixes SP2 and SP3 allowed isolation of both the ca. 71 kbp as well as the ca. 180 kbp plasmids. The plasmids of ca. 71 kbp in this study that were identified as IncFII replicon types also possessed the mating pair system MPF_I_ while the larger plasmids of ca. 180 bp that were identified as IncFIB plasmids belonged to the mating pair system MPF_F_ and therefore all these plasmids were considered to be transferable by conjugation (Table 1). All small plasmids except for the Col440II plasmid of the transconjugant X/SP2-CαI/13 were determined to be non-mobilizable by Mob-suite.

### Sprout 16S rRNA-gene microbiome analysis

**Figure 3:**
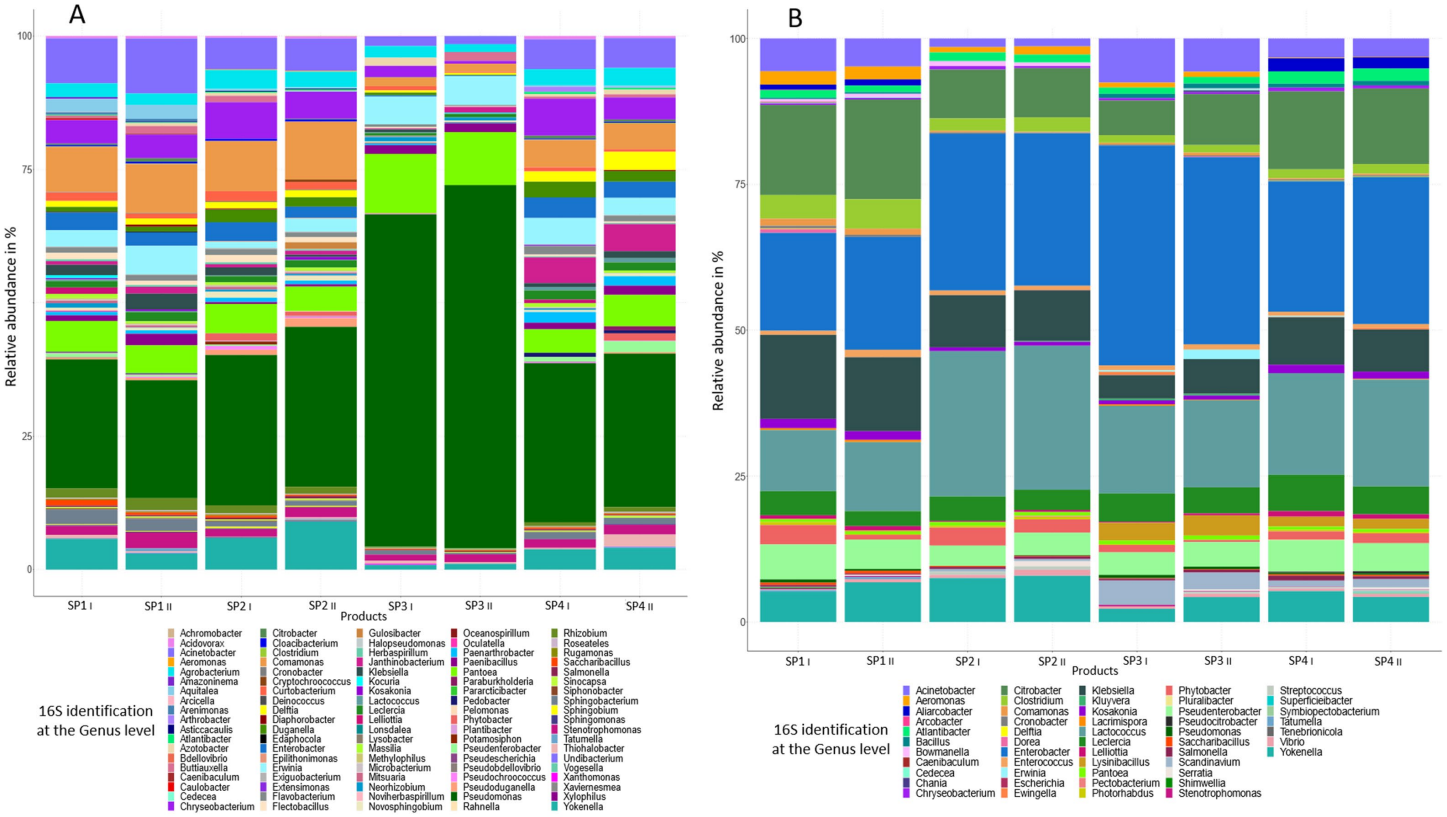
Bacterial community composition and relative abundance at the genus level in non-enriched sprouts **(A)** and enriched sprout samples **(B)**. Each set included four sprout samples obtained on different dates from retail, with metagenomic analysis performed in technical duplicates.

MinION sequencing was used to sequence the almost complete 16S rRNA genes of the bacterial microbiota of the four sprout samples. This was done to characterize the potential plasmid donors present in the sprout microbiota that may have possibly transferred their plasmids during the exogenous plasmid capture experiment. Figure 3A shows the high diversity of the bacteria in the microbiota of the non-enriched sprout samples, where as much as ca. 100 different genera could be detected. It could, however, be observed that not every genus was present in all the different sprout samples. The technical replicates were observed to show a high similarity regarding the different genera detected and in their abundances. All sprout samples were dominated by the genus *Pseudomonas*, whereas the genera *Acinetobacter, Comamonas, Chryseobacterium, Yokenella, Erwinia* and *Pantoea* were also among the most commonly occurring genera.

**Figure 4:**
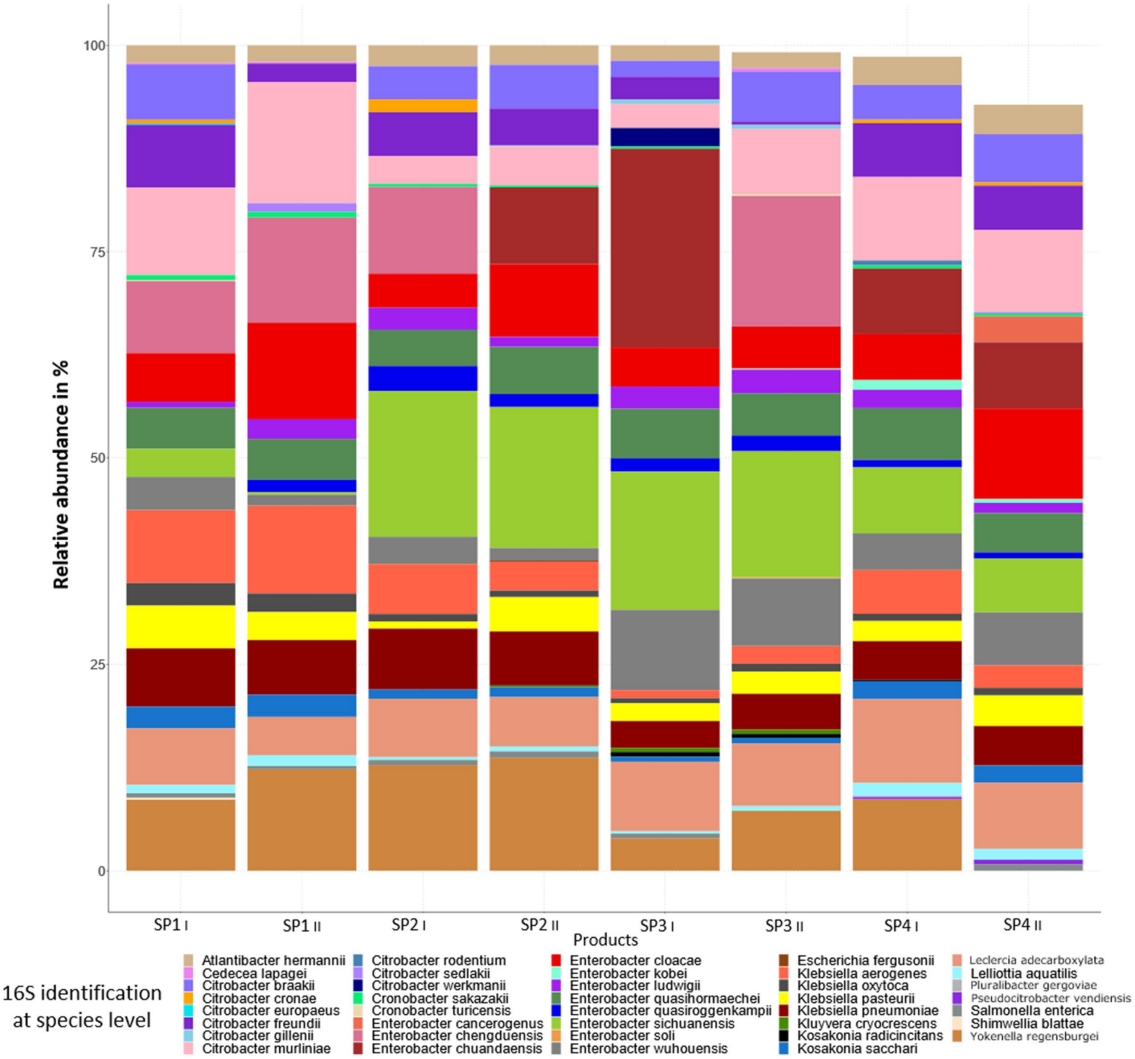
Bacterial community composition and relative abundance at the species level in enriched sprout samples.

Figure 3B shows the relative abundances of genera present in the sprout samples after enrichment in peptone water for 20–24 h at 37°C. Enrichment clearly led to a decrease in the bacterial diversity, as after enrichment only ca. 50 genera were detected. Also, in this case not all genera were present in the different sprout samples (Figure 3B). In the enriched samples genera were detected which were probably below the detection limit in the non-enriched sprout samples. These included *Bacillus, Bowmanella, Enterococcus, Scandinavium, Serratia* and *Vibrio*. The genera with the highest relative abundances in all samples were *Enterobacter*, followed by *Citrobacter, Lactococcus* and *Klebsiella*. The genus *Escherichia* was detected only in one of the duplicate SP2 sprout samples at a relative abundance of 0.07%. The enriched samples thus showed a relatively large proportion of *Enterobacteriaceae*, which differed only marginally between the different sprout samples and technical replicates. A species level analysis of the Enterobacteriaceae also showed among other *Enterobacteriaceae* the presence of *Enterobacter cloacae*, *E. ludwigii*, *E. kobei*, *Citrobacter werkmanii*, *C. freundii*, *K. oxytoca* and *K. pneumonia* (Figure 4). These bacteria were previously isolated from fresh produce in Germany and found to contain large antibiotic resistance plasmids (Stein et al., 2024) and thus could have possibly also served as the donors for antibiotic resistance plasmids identified in this study.

## Discussion

Sprouts were used for exogenous plasmid capture in this study because these food products were found to be typically colonized by a high number of bacteria, including *Enterobacteriaceae* (Boehme et al., 2004; Abadias et al., 2008; Berthold-Pluta et al., 2017; Keshri et al., 2019), which frequently included both pathogenic (Taormina et al., 1999; Buchholz et al., 2011) and antibiotic-resistant strains (Boehme et al., 2004; Freitag et al., 2018; Huizinga et al., 2018). In this study, it could be shown that transfer of antibiotic-resistance plasmids could indeed occur directly on sprout products at 37°C, as was tested using an exogenous plasmid-capture approach. Sprouts are generally not produced on the field, but are rather grown indoors under warm and moist conditions. Previously, transfer of antibiotic resistance plasmids was shown to occur already during sprouting of the seeds as well as subsequent growth, using a donor/recipient system (Luo and Matthews, 2023) or using a donor which transferred its plasmid DNA to bacteria of the sprout microbiota (Molbak et al., 2003).

For investigations on the dissemination of antibiotic resistance genes along the farm-to-fork route, the determinations on the occurrence of conjugative antibiotic resistance plasmids in bacteria on plant products becomes important. Blau et al. (2018) studied the occurrence of conjugative plasmids on plant products including salad, arugula and coriander also using exogenous plasmid capture. For this, they enriched bacteria from these products before using them as donor strains for exogenous plasmid capture in filter mating experiments. Using this method, Blau et al. (2018) isolated tetracycline-resistant transconjugants with a transfer rate of approx. 10^−4^ in mixed salad and 10^−9^ in arugula. Our study differed from Blau et al. (2018) in that we used a 1:10 ratio of enriched bacterial sample to recipient, rather than 1:40, and performed conjugation at 37°C instead of 28°C. Furthermore, in our study, the bacteria were not mated on filters for exogenous plasmid capture. Instead, the mating process was carried out directly on the sprout product. This study also showed that exogenous plasmid captures by the *E. coli* CV601 recipient strain was possible using bacteria from sprouts as plasmid donor even without preforming a prior enrichment. The method therefore seems to be useful for testing the presence of transferable plasmids bearing antibiotic-resistance genes directly within food environments. The *E. coli* CV601 strain was already used for conjugation experiments or exogenous plasmids also in other studies (Heuer et al., 2012; Wolters et al., 2014; Marathe et al., 2021). However, it should be remembered that the strain also influences the spectrum of plasmids that can be transferred (Sheppard et al., 2020; Benz et al., 2021), so that probably not all mobile resistance plasmids present in the mobilome of sprouts would be detected. As an *E. coli* strain was used as recipient, the focus of the study was clearly on capture of plasmids from *Enterobacteriaceae*; however, our 16S rRNA metagenomic data showed that pseudomonads were the dominating genus occurring in these products (Figure 3A). Thus, to address this finding, a *Pseudomonas* (*P*.) recipient strain such as *P. putida* UWC1 (Smalla et al., 2000) could be used in further studies to determine the transferable plasmids present also in these bacteria.

An interesting finding of this exogenous plasmid capture study was that transconjugants were obtained not with cefotaxime, but with tetracycline. Cefotaxime antibiotic was used as selective agent in agar plates to capture plasmids with the ESBL phenotype, i.e., beta-lactamases with an extended activity spectrum. These are encoded by genes such as *bla*CTX, which are often located on transferable plasmids that have previously been shown to present in bacteria from fresh produce (Wang et al., 2013; Branger et al., 2018; Wang et al., 2022). Sprouts, in particular, have previously been shown to become contaminated with *Enterobacteriaceae* via several routes, and some of these contaminating *Enterobacteriaceae* were found to harbor ESBL genes such as *bla*CTX-M and *bla*SHV (Reuland et al., 2014; Margot et al., 2016; Huizinga et al., 2018; Stein et al., 2024). However, the incidences of bacteria harboring these ESBL genes was reported to be only 2.9% for sprout samples in Switzerland (of n = 102 investigated in total) (Margot et al., 2016), and 25% of 131 bean sprout samples from the Netherlands (Huizinga et al., 2018). A German study focusing on foods of different origin found ESBL bacteria in 0.3% of vegetable samples (Kaesbohrer et al., 2019). This low incidence and the relatively short sampling period in which sprout products were obtained from retail and analyzed may be the reasons for the fact that no cefotaxime-resistant transconjugants could be isolated in this study.

Blau et al. (2018) isolated a total of 91 tetracycline-resistant transconjugants after enrichment of bacteria from coriander, arugula and mixed salads, of which 15 were selected for further characterization. These plasmids exhibited different replicon types, i.e., IncFIB, IncFII, IncP and IncI, among which IncFIB and IncFII were the most commonly occurring (Blau et al., 2018). Most of the plasmid replicon types in this study could also be identified as IncFIB and IncFII replicons, whereas IncI and IncP replicons detected by Blau et al. (2018) could not be identified. Moreover, in our study mobilizable Col plasmids could be determined to occur together with the IncFIB(K) and the IncFIB/IncFII(K) multireplicon plasmids. One reason why these were not detected in the study by Blau et al. (2018) may be, that these authors used PCR for replicon typing and that there were no PCR primers available for detecting the Col replicons (Carattoli et al., 2005; Blau et al., 2018). As this study, on the other hand, was based on whole genome sequencing, the plasmids could be searched for replicon types using PlasmidFinder. The study of Barry et al. (2019) showed that the cryptic and mobilizable Col440I, which was identified also in this study, could be co-transferred by larger antibiotic resistance plasmids at a high rate. This could explain why such small Col(pHAD28), Col440I and Col440II plasmids were captured together with larger plasmids in this study, even though these Col plasmids did not encode any resistance genes. The Col plasmids Col440I and Col(pHAD28) were determined by MOB-suite to be non-mobilizable. However, as the combination of both these small plasmids was detected together with the IncFIB(K) plasmids in the sprout samples SP2, SP3 and SP4, hypothetically it can be assumed that these are indeed mobilized by the conjugative apparatus of the IncFIB(K) plasmids.

In this study very similar or even identical antibiotic resistance plasmids were captured over a rather short sampling period of ca. 3 weeks. In the sprout sample SP1, only the smaller antibiotic resistance plasmids ca. 71 kbp was captured, while in sample SP4, only the larger antibiotic resistance plasmids of ca. 180 kbp were obtained. The sprout samples SP2 and SP3 contained both the smaller 71 kbp and larger 180 kbp plasmids. The smaller plasmid possessing antibiotic resistance genes, however, was found more often and showed a 100% identity in size (71,155 bp) between the different sprout samples, while the 180 kbp plasmids showed differences in size (between 157,683 to 192,360 bp). This indicated that the plasmids obtained from similar sprout production batches were probably clonally related, and that adapted strains may be present either in the sprout seeds or the sprout production environment. Huizinga et al. (2018) isolated ESBL producing *K. pneumoniae*, *K. oxytoca*, *K.* var*iicola* and *En. Cloacae* from bean sprouts. These authors showed that over time, the isolates were genetically quite distant, while isolates from samples closely matched in time were frequently clonally related. This indicated a batch contamination, which is similar to the findings of this study. The plasmid of transconjugant X/SP2-CαI/13 showed similarity to the other captured IncFIB plasmids, however, this plasmid was larger and possessed the apart from the *tet*(D) resistance gene also the *sul*1 and *aad*A1 resistance genes (Table 1). Furthermore, PlasmidFinder detected not only the IncFIB(K) but also the IncFII(K) replicon type in this plasmid. Furthermore, this transconjugant was the only one to possess also a further Col440II replicon plasmid. Thus, it can be assumed that not only horizontal gene transfer but also recombination took place, leading to a plasmid with the combined IncFIB(K) and IncFII(K) replicons. However, it was not possible to determine if this hypothetical recombination would have occurred in the production environment or in the experiment itself. This would have required a parallel study where antibiotic resistant strains from sprouts were isolated on tetracycline-containing medium and identified, which was not aimed for in this study.

The bacteria from the non-enriched sprout samples represented the microbiota of the product after buying at retail (cold storage at supermarket). The 16S rRNA amplicon sequencing clearly showed a high bacterial diversity in these samples (Figure 3A). High bacterial diversity was already noted in a previous study, in which alfalfa and mung bean sprouts from two different producers showed 53 to 107 different genera (alfalfa bean sprouts) or 36 to 61 different genera (mung bean sprouts; Keshri et al., 2019). As in our study, *Pseudomonas* spp. were also found to predominate in the microbiota of alfalfa and mung bean sprouts in the study of Keshri et al. (2019), as well as in a study on the microbiota of radish sprouts by Asakura et al. (2016). In our study, it could also be shown by using the complete 16S rRNA gene amplicon sequencing data that the enriched sprout samples contained high relative abundances of the different *Enterobacteriaceae* such as *Citrobacter, Klebsiella*, *Enterobacter* and *Escherichia,* which occurred at proportions between 43 to 48% in the different samples analyzed (Figure 4). Together, the *Enterobacteriaceae* occurred at relative abundances of above 50% in the different sprout samples. As none of the strains occurring in the sprout samples were further analyzed, it was impossible to make conclusions as to which of the specific genera the exogenously captured plasmid found in this study originated. Our previous study on antibiotic-resistant bacteria from fresh produce from retail in Germany, however, also showed that IncFIB and IncFII plasmids harboring either *tet*(A) or *tet*(D) antibiotic resistance genes occurred among *Enterobacteriaceae* such as *Enterobacter* (*En.*) *cloacae*, *En. ludwigii*, *E. kobei*, *Citrobacter* (*C*.) *werkmanii*, *C. freundii*, *Klebsiella* (*K.*) *oxytoca* and *K. pneumoniae* (Stein et al., 2024).

Our results thus indicate that E*nterobacteriaceae* on fresh produce may harbor plasmids with replicons that allow replication among this group of bacteria (IncFIB and IncFII) and which are mobilizable. These therefore seem to readily be transmitted between these bacteria as they are predicted to be mobilizable by Mob-suite and indeed exogenous plasmid capture experiments show that transfer can occur directly on the product without the need for bacterial enrichment. These plasmids contain tetracycline resistance genes as well as sulfonamide resistance genes and some studies have shown the presence of also ESBL encoding genes on plasmids in *Enterobacteriaceae* in produce or sprouts (Reuland et al., 2014; Freitag et al., 2018; Huizinga et al., 2018). This suggests that produce may serve as a vehicle for transmission of antibiotic resistant bacteria and their genes to humans if these products are eaten raw and survive the gastrointestinal transit. Efforts should therefore be directed to the prevention and monitoring of the occurrence of such antibiotic-resistant bacteria in produce.

## Data Availability

The unique extrachromosomal DNA sequences were deposited in the GenBank/ENA/DDBJ databases under the accession numbers listed in Table 1.

## Footnotes

^1^ https://ggdc.dsmz.de/ggdc.php

^2^https://cge.food.dtu.dk/services/PlasmidFinder/; v. 2.1

^3^http://genepi.food.dtu.dk/resfinder; v. 4.6.0

^4^ https://phaster.ca/

## References

[ref1] Abadias M. Usall J. Anguera M. Solsona C. Vinas I. (2008). Microbiological quality of fresh, minimally-processed fruit and vegetables, and sprouts from retail establishments. Int. J. Food Microbiol. 123, 121–129. doi: 10.1016/j.ijfoodmicro.2007.12.013, PMID: 18237811

[ref2] Altschul S. F. Gish W. Miller W. Myers E. W. Lipman D. J. (1990). Basic local alignment search tool. J. Mol. Biol. 215, 403–410. doi: 10.1016/s0022-2836(05)80360-2, PMID: 2231712

[ref3] Aminov R. I. (2011). Horizontal gene exchange in environmental microbiota. Front. Microbiol. 2:158. doi: 10.3389/fmicb.2011.00158, PMID: 21845185 PMC3145257

[ref4] Antimicrobial Resistance C. (2022). Global burden of bacterial antimicrobial resistance in 2019: a systematic analysis. Lancet 399, 629–655. doi: 10.1016/S0140-6736(21)02724-0, PMID: 35065702 PMC8841637

[ref5] Arndt D. Grant J. R. Marcu A. Sajed T. Pon A. Liang Y.. (2016). PHASTER: a better, faster version of the PHAST phage search tool. Nucleic Acids Res. 44, W16–W21. doi: 10.1093/nar/gkw387, PMID: 27141966 PMC4987931

[ref6] Asakura H. Tachibana M. Taguchi M. Hiroi T. Kurazono H. Makino S. I.. (2016). Seasonal and growth-dependent dynamics of bacterial Community in Radish Sprouts. J. Food Saf. 36, 392–401. doi: 10.1111/jfs.12256

[ref7] Barry K. E. Wailan A. M. Sheppard A. E. Crook D. Vegesana K. Stoesser N.. (2019). Don't overlook the little guy: an evaluation of the frequency of small plasmids co-conjugating with larger carbapenemase gene containing plasmids. Plasmid 103, 1–8. doi: 10.1016/j.plasmid.2019.03.005, PMID: 30928702

[ref8] Benz F. Huisman J. S. Bakkeren E. Herter J. A. Stadler T. Ackermann M.. (2021). Plasmid- and strain-specific factors drive variation in ESBL-plasmid spread in vitro and in vivo. ISME J. 15, 862–878. doi: 10.1038/s41396-020-00819-4, PMID: 33149210 PMC8026971

[ref9] Berthold-Pluta A. Garbowska M. Stefanska I. Pluta A. (2017). Microbiological quality of selected ready-to-eat leaf vegetables, sprouts and non-pasteurized fresh fruit-vegetable juices including the presence of Cronobacter spp. Food Microbiol. 65, 221–230. doi: 10.1016/j.fm.2017.03.005, PMID: 28400006

[ref10] Blau K. Bettermann A. Jechalke S. Fornefeld E. Vanrobaeys Y. Stalder T.. (2018). The transferable Resistome of produce. MBio 9:e01300. doi: 10.1128/mBio.01300-18 30401772 PMC6222124

[ref11] Blau K. Jechalke S. Smalla K. (2020). Detection, isolation, and characterization of plasmids in the environment. Methods Mol. Biol. 2075, 39–60. doi: 10.1007/978-1-4939-9877-7_3 31584153

[ref12] Boehme S. Werner G. Klare I. Reissbrodt R. Witte W. (2004). Occurrence of antibiotic-resistant enterobacteria in agricultural foodstuffs. Mol. Nutr. Food Res. 48, 522–531. doi: 10.1002/mnfr.200400030, PMID: 15538714

[ref13] Borges A. S. G. Basu M. Brinks E. Bang C. Cho G. S. Baines J. F.. (2023). Fast identification method for screening Bacteria from Faecal samples using Oxford Nanopore technologies MinION sequencing. Curr. Microbiol. 80:101. doi: 10.1007/s00284-023-03201-7, PMID: 36759384 PMC9911510

[ref14] Branger C. Ledda A. Billard-Pomares T. Doublet B. Fouteau S. Barbe V.. (2018). Extended-spectrum beta-lactamase-encoding genes are spreading on a wide range of *Escherichia coli* plasmids existing prior to the use of third-generation cephalosporins. Microb Genom 4:203. doi: 10.1099/mgen.0.000203, PMID: 30080134 PMC6202452

[ref15] Buchholz U. Bernard H. Werber D. Bohmer M. M. Remschmidt C. Wilking H.. (2011). German outbreak of *Escherichia coli* O104:H4 associated with sprouts. N. Engl. J. Med. 365, 1763–1770. doi: 10.1056/NEJMoa1106482, PMID: 22029753

[ref16] Bundesinstitut für Risikobewertung (2020). *Escherichia coli* in flour – sources, risks and prevention. BfR Opin. 20:303. doi: 10.17590/20200120-102303

[ref17] Callejon R. M. Rodriguez-Naranjo M. I. Ubeda C. Hornedo-Ortega R. Garcia-Parrilla M. C. Troncoso A. M. (2015). Reported foodborne outbreaks due to fresh produce in the United States and European Union: trends and causes. Foodborne Pathog. Dis. 12, 32–38. doi: 10.1089/fpd.2014.1821, PMID: 25587926

[ref18] Canning M. Birhane M. G. Dewey-Mattia D. Lawinger H. Cote A. Gieraltowski L.. (2023). Salmonella outbreaks linked to beef, United States, 2012-2019. J. Food Prot. 86:100071. doi: 10.1016/j.jfp.2023.100071, PMID: 37028195 PMC10966622

[ref19] Carattoli A. Bertini A. Villa L. Falbo V. Hopkins K. L. Threlfall E. J. (2005). Identification of plasmids by PCR-based replicon typing. J. Microbiol. Methods 63, 219–228. doi: 10.1016/j.mimet.2005.03.018, PMID: 15935499

[ref20] Cho G. S. Stein M. Fiedler G. Igbinosa E. O. Koll L. P. Brinks E.. (2021). Polyphasic study of antibiotic-resistant enterobacteria isolated from fresh produce in Germany and description of *Enterobacter vonholyi* sp. nov. isolated from marjoram and *Enterobacter dykesii* sp. nov. isolated from mung bean sprout. Syst. Appl. Microbiol. 44:126174. doi: 10.1016/j.syapm.2020.126174, PMID: 33370657

[ref21] Chris Baylis M. U. Joosten H. Davies A. (2011). *The Enterobacteriaceae and their significance to the food industry*. ILSI Europe Report Series.

[ref22] Clinical and Laboratory Standards Institute. (2022). *Performance Standards for Antimicrobial Susceptibility Testing*. 32nd Edition. Clinical and Laboratory Standards Institute).

[ref23] Collignon P. J. McEwen S. A. (2019). One health-its importance in helping to better control Antimicrobial Resistance. Trop med. Infect. Dis. 4:22. doi: 10.3390/tropicalmed4010022, PMID: 30700019 PMC6473376

[ref24] Fiedler G. Kabisch J. Bohnlein C. Huch M. Becker B. Cho G. S.. (2017). Presence of human pathogens in produce from retail Markets in Northern Germany. Foodborne Pathog. Dis. 14, 502–509. doi: 10.1089/fpd.2016.2258, PMID: 28594569

[ref25] Frank J. A. Reich C. I. Sharma S. Weisbaum J. S. Wilson B. A. Olsen G. J. (2008). Critical evaluation of two primers commonly used for amplification of bacterial 16S rRNA genes. Appl. Environ. Microbiol. 74, 2461–2470. doi: 10.1128/AEM.02272-07, PMID: 18296538 PMC2293150

[ref26] Frank C. Werber D. Cramer J. P. Askar M. Faber M. an der Heiden M.. (2011). Epidemic profile of Shiga-toxin-producing *Escherichia coli* O104:H4 outbreak in Germany. N. Engl. J. Med. 365, 1771–1780. doi: 10.1056/NEJMoa1106483, PMID: 21696328

[ref27] Freitag C. Michael G. B. Li J. Kadlec K. Wang Y. Hassel M.. (2018). Occurrence and characterisation of ESBL-encoding plasmids among *Escherichia coli* isolates from fresh vegetables. Vet. Microbiol. 219, 63–69. doi: 10.1016/j.vetmic.2018.03.028, PMID: 29778206

[ref28] Herman K. M. Hall A. J. Gould L. H. (2015). Outbreaks attributed to fresh leafy vegetables, United States, 1973-2012. Epidemiol. Infect. 143, 3011–3021. doi: 10.1017/S0950268815000047, PMID: 25697407 PMC4591532

[ref29] Heuer H. Binh C. T. Jechalke S. Kopmann C. Zimmerling U. Krogerrecklenfort E.. (2012). IncP-1epsilon plasmids are important vectors of antibiotic Resistance genes in agricultural systems: diversification driven by class 1 Integron gene cassettes. Front. Microbiol. 3:2. doi: 10.3389/fmicb.2012.00002, PMID: 22279444 PMC3260659

[ref30] Heuer H. Krogerrecklenfort E. Wellington E. M. Egan S. van Elsas J. D. van Overbeek L.. (2002). Gentamicin resistance genes in environmental bacteria: prevalence and transfer. FEMS Microbiol. Ecol. 42, 289–302. doi: 10.1111/j.1574-6941.2002.tb01019.x, PMID: 19709289

[ref31] Heuer H. Schmitt H. Smalla K. (2011). Antibiotic resistance gene spread due to manure application on agricultural fields. Curr. Opin. Microbiol. 14, 236–243. doi: 10.1016/j.mib.2011.04.009, PMID: 21546307

[ref32] Huizinga P. Schrauwen E. Garcia-Cobos S. Willemsen I. Verhulst C. Friedrich A. W.. (2018). Extended-spectrum beta-lactamase producing Enterobacteriaceae (ESBL-E) isolated from bean sprouts in the Netherlands. PLoS One 13:e0203338. doi: 10.1371/journal.pone.0203338, PMID: 30161223 PMC6117087

[ref33] Iseppi R. de Niederhausern S. Bondi M. Messi P. Sabia C. (2018). Extended-Spectrum beta-lactamase, AmpC, and MBL-producing gram-negative Bacteria on fresh vegetables and ready-to-eat salads sold in local markets. Microb. Drug Resist. 24, 1156–1164. doi: 10.1089/mdr.2017.0198, PMID: 29451428

[ref1002] Jechalke S. Heuer H. Siemens J. Amelung W. Smalla K. (2014). Fate and effects of veterinary antibiotics in soil. Trends Microbiol. 22, 536–546. doi: 10.1016/j.tim.2014.05.005, PMID: 24950802

[ref34] Jian Z. Zeng L. Xu T. Sun S. Yan S. Yang L.. (2021). Antibiotic resistance genes in bacteria: occurrence, spread, and control. J. Basic Microbiol. 61, 1049–1070. doi: 10.1002/jobm.202100201, PMID: 34651331

[ref35] Kaesbohrer A. Bakran-Lebl K. Irrgang A. Fischer J. Kampf P. Schiffmann A.. (2019). Diversity in prevalence and characteristics of ESBL/pAmpC producing *E. coli* in food in Germany. Vet. Microbiol. 233, 52–60. doi: 10.1016/j.vetmic.2019.03.025, PMID: 31176413

[ref36] Keshri J. Krouptiski Y. Abu-Fani L. Achmon Y. Bauer T. S. Zarka O.. (2019). Dynamics of bacterial communities in alfalfa and mung bean sprouts during refrigerated conditions. Food Microbiol. 84:103261. doi: 10.1016/j.fm.2019.103261, PMID: 31421775

[ref37] Kläui A. Bütikofer U. Naskova J. Wagner E. Marti E. (2024). Fresh produce as a reservoir of antimicrobial resistance genes: a case study of Switzerland. Sci. Total Environ. 907:167671. doi: 10.1016/j.scitotenv.2023.167671, PMID: 37813266

[ref38] Krych L. Castro-Mejia J. L. Forero-Junco L. M. Moesby D. N. Mikkelsen M. B. Rasmussen M. A.. (2019). DNA enrichment and tagmentation method for species-level identification and strain-level differentiation using ON-rep-seq. Commun. Biol. 2:369. doi: 10.1038/s42003-019-0617-x, PMID: 31633060 PMC6787052

[ref39] Kumar S. Mukherjee M. M. Varela M. F. (2013). Modulation of bacterial multidrug Resistance efflux pumps of the major facilitator superfamily. Int. J. Bacteriol. 2013:204141, 1–15. doi: 10.1155/2013/204141, PMID: 25750934 PMC4347946

[ref40] Kuske C. R. Barns S. M. Grow C. C. Merrill L. Dunbar J. (2006). Environmental survey for four pathogenic bacteria and closely related species using phylogenetic and functional genes. J. Forensic Sci. 51, 548–558. doi: 10.1111/j.1556-4029.2006.00131.x, PMID: 16696701

[ref41] Leff J. W. Fierer N. (2013). Bacterial communities associated with the surfaces of fresh fruits and vegetables. PLoS One 8:e59310. doi: 10.1371/journal.pone.0059310, PMID: 23544058 PMC3609859

[ref42] Lima T. Domingues S. Da Silva G. J. (2020). Manure as a potential hotspot for antibiotic Resistance dissemination by horizontal gene transfer events. Vet. Sci. 7:110. doi: 10.3390/vetsci7030110, PMID: 32823495 PMC7558842

[ref43] Luo X. Matthews K. R. (2023). The conjugative transfer of plasmid-mediated mobile colistin resistance gene, mcr-1, to *Escherichia coli* O157:H7 and *Escherichia coli* O104:H4 in nutrient broth and in mung bean sprouts. Food Microbiol. 111:104188. doi: 10.1016/j.fm.2022.104188, PMID: 36681389

[ref44] Lynch M. F. Tauxe R. V. Hedberg C. W. (2009). The growing burden of foodborne outbreaks due to contaminated fresh produce: risks and opportunities. Epidemiol. Infect. 137, 307–315. doi: 10.1017/S0950268808001969 19200406

[ref45] Marathe N. P. Svanevik C. S. Ghavidel F. Z. Grevskott D. H. (2021). First report of mobile tigecycline resistance gene tet(X4)-harbouring multidrug-resistant *Escherichia coli* from wastewater in Norway. J. Glob. Antimicrob. Resist. 27, 37–40. doi: 10.1016/j.jgar.2021.07.019, PMID: 34371242

[ref46] Margot H. Ebner R. Peterhans S. Stephan R. (2016). Occurrence of *Salmonella*, *L. monocytogenes*, Shigatoxin-producing *E. coli* and ESBL-producing Enterobacteriaceae in sprout samples collected from the Swiss market. J. Verbrauch. Lebensm. 11, 155–157. doi: 10.1007/s00003-015-1003-3

[ref47] McEwen S. A. Collignon P. J. (2018). Antimicrobial Resistance: a one health perspective. Microbiol. Spectr. 6:2017. doi: 10.1128/microbiolspec.ARBA-0009-2017, PMID: 29600770 PMC11633550

[ref48] Meier-Kolthoff J. P. Klenk H.-P. Göker M. (2014). Taxonomic use of DNA G+C content and DNA–DNA hybridization in the genomic age. Int. J. Syst. Evol. Microbiol. 64, 352–356. doi: 10.1099/ijs.0.056994-0, PMID: 24505073

[ref49] Molbak L. Licht T. R. Kvist T. Kroer N. Andersen S. R. (2003). Plasmid transfer from *Pseudomonas putida* to the indigenous bacteria on alfalfa sprouts: characterization, direct quantification, and in situ location of transconjugant cells. Appl. Environ. Microbiol. 69, 5536–5542. doi: 10.1128/AEM.69.9.5536-5542.2003, PMID: 12957943 PMC194921

[ref50] Nuesch-Inderbinen M. T. Funk J. Cernela N. Tasara T. Klumpp J. Schmidt H.. (2015). Prevalence of subtilase cytotoxin-encoding subAB variants among Shiga toxin-producing *Escherichia coli* strains isolated from wild ruminants and sheep differs from that of cattle and pigs and is predominated by the new allelic variant subAB2-2. Int. J. Med. Microbiol. 305, 124–128. doi: 10.1016/j.ijmm.2014.11.009, PMID: 25488108

[ref51] Olaimat A. N. Holley R. A. (2012). Factors influencing the microbial safety of fresh produce: a review. Food Microbiol. 32, 1–19. doi: 10.1016/j.fm.2012.04.016, PMID: 22850369

[ref52] Pasqua M. Bonaccorsi di Patti M. C. Fanelli G. Utsumi R. Eguchi Y. Trirocco R.. (2021). Host - bacterial pathogen communication: the wily role of the multidrug efflux pumps of the MFS family. Front. Mol. Biosci. 8:723274. doi: 10.3389/fmolb.2021.723274, PMID: 34381818 PMC8350985

[ref53] Ragaert P. Devlieghere F. Debevere J. (2007). Role of microbiological and physiological spoilage mechanisms during storage of minimally processed vegetables. Postharvest Biol. Technol. 44, 185–194. doi: 10.1016/j.postharvbio.2007.01.001

[ref54] Reuland E. A. Al Naiemi N. Raadsen S. A. Savelkoul P. H. Kluytmans J. A. Vandenbroucke-Grauls C. M. (2014). Prevalence of ESBL-producing Enterobacteriaceae in raw vegetables. Eur. J. Clin. Microbiol. Infect. Dis. 33, 1843–1846. doi: 10.1007/s10096-014-2142-7, PMID: 24848131 PMC4182617

[ref55] Robertson J. Nash J. H. E. (2018). MOB-suite: software tools for clustering, reconstruction and typing of plasmids from draft assemblies. Microb. Genom. 4:206. doi: 10.1099/mgen.0.000206, PMID: 30052170 PMC6159552

[ref56] Rodriguez-Perez H. Ciuffreda L. Flores C. (2021). NanoCLUST: a species-level analysis of 16S rRNA nanopore sequencing data. Bioinformatics 37, 1600–1601. doi: 10.1093/bioinformatics/btaa900, PMID: 33079990

[ref57] Schwaiger K. Helmke K. Holzel C. S. Bauer J. (2011). Antibiotic resistance in bacteria isolated from vegetables with regards to the marketing stage (farm vs. supermarket). Int. J. Food Microbiol. 148, 191–196. doi: 10.1016/j.ijfoodmicro.2011.06.001, PMID: 21700353

[ref58] Self J. L. Conrad A. Stroika S. Jackson A. Whitlock L. Jackson K. A.. (2019). Multistate outbreak of Listeriosis associated with packaged leafy green salads, United States and Canada, 2015-2016. Emerg. Infect. Dis. 25, 1461–1468. doi: 10.3201/eid2508.180761, PMID: 31310227 PMC6649349

[ref59] Sharapov U. M. Wendel A. M. Davis J. P. Keene W. E. Farrar J. Sodha S.. (2016). Multistate outbreak of *Escherichia coli* O157:H7 infections associated with consumption of fresh spinach: United States, 2006. J. Food Prot. 79, 2024–2030. doi: 10.4315/0362-028X.JFP-15-556, PMID: 28221950

[ref60] Sheppard R. J. Beddis A. E. Barraclough T. G. (2020). The role of hosts, plasmids and environment in determining plasmid transfer rates: a meta-analysis. Plasmid 108:102489. doi: 10.1016/j.plasmid.2020.102489, PMID: 31926878

[ref61] Smalla K. Heuer H. Gotz A. Niemeyer D. Krogerrecklenfort E. Tietze E. (2000). Exogenous isolation of antibiotic resistance plasmids from piggery manure slurries reveals a high prevalence and diversity of IncQ-like plasmids. Appl. Environ. Microbiol. 66, 4854–4862. doi: 10.1128/AEM.66.11.4854-4862.2000, PMID: 11055935 PMC92391

[ref62] Stein M. Brinks E. Loop J. Habermann D. Cho G. S. Franz C. (2024). Antibiotic resistance plasmids in Enterobacteriaceae isolated from fresh produce in northern Germany. Microbiol. Spectr. 12:e0036124. doi: 10.1128/spectrum.00361-24, PMID: 39287384 PMC11537058

[ref63] Stothard P. Wishart D. S. (2005). Circular genome visualization and exploration using CGView. Bioinformatics 21, 537–539. doi: 10.1093/bioinformatics/bti054, PMID: 15479716

[ref64] Taormina P. J. Beuchat L. R. Slutsker L. (1999). Infections associated with eating seed sprouts: an international concern. Emerg. Infect. Dis. 5, 626–634. doi: 10.3201/eid0505.990503, PMID: 10511518 PMC2627711

[ref65] Tatusova T. DiCuccio M. Badretdin A. Chetvernin V. Nawrocki E. P. Zaslavsky L.. (2016). NCBI prokaryotic genome annotation pipeline. Nucleic Acids Res. 44, 6614–6624. doi: 10.1093/nar/gkw569, PMID: 27342282 PMC5001611

[ref66] Vestrheim D. F. Lange H. Nygard K. Borgen K. Wester A. L. Kvarme M. L.. (2016). Are ready-to-eat salads ready to eat? An outbreak of Salmonella Coeln linked to imported, mixed, pre-washed and bagged salad, Norway, November 2013. Epidemiol. Infect. 144, 1756–1760. doi: 10.1017/S0950268815002769, PMID: 26586305 PMC9150709

[ref67] von Wintersdorff C. J. Penders J. van Niekerk J. M. Mills N. D. Majumder S. van Alphen L. B.. (2016). Dissemination of Antimicrobial Resistance in microbial ecosystems through horizontal gene transfer. Front. Microbiol. 7:173. doi: 10.3389/fmicb.2016.00173 26925045 PMC4759269

[ref68] Wang Y. Batra A. Schulenburg H. Dagan T. (2022). Gene sharing among plasmids and chromosomes reveals barriers for antibiotic resistance gene transfer. Philos. Trans. R. Soc. Lond. Ser. B Biol. Sci. 377:20200467. doi: 10.1098/rstb.2020.0467, PMID: 34839702 PMC8628082

[ref69] Wang J. Stephan R. Karczmarczyk M. Yan Q. Hachler H. Fanning S. (2013). Molecular characterization of Bla ESBL-harboring conjugative plasmids identified in multi-drug resistant *Escherichia coli* isolated from food-producing animals and healthy humans. Front. Microbiol. 4:188. doi: 10.3389/fmicb.2013.00188, PMID: 23874325 PMC3708134

[ref70] Wolters B. Kyselkova M. Krogerrecklenfort E. Kreuzig R. Smalla K. (2014). Transferable antibiotic resistance plasmids from biogas plant digestates often belong to the IncP-1epsilon subgroup. Front. Microbiol. 5:765. doi: 10.3389/fmicb.2014.00765, PMID: 25653641 PMC4301011

